
JOURNAL INFORMATION
==============================
NLM Title Abbreviation: ScientificWorldJournal
Journal ID: ScientificWorldJournal
NLM Journal ID: 101131163
Journal ID: TSWJ

The Scientific World Journal

ISSN: 2356-6140
EISSN: 1537-744X
Publisher: Wiley

ARTICLE INFORMATION
==============================
PMCID: PMC6084422
PMID: 30147584
DOI: 10.1100/tsw.2001.119
Article ID: 570408
Article version: 1
Subjects: Short Report

Determination of the Effects of Camptothecin on Cell Cycling and Apoptosis in Human Leukaemic Cell Lines

Cox Anne 1 *
Bradbury Dawn A. 2
Simmons Tracy D. 1
Slater Kevin J. 1
Crouch Sharon P. M. 1
1 LumiTech (UK) Ltd, Nottingham Business Park, City Link, Nottingham NG2 4LA, UK
2 Clinical Sciences, Nottingham University, Nottingham, UK
*Anne Cox: a.cox@lumitech.co.uk
Publication date: 2001
Electronic publication date: 2001 May 1
Volume: 1
First page: 105
Last page: 105
Copyright: Copyright © 2001 Anne Cox et al.
Copyright year: 2001
License: This is an open access article distributed under the Creative Commons Attribution License, which permits unrestricted use, distribution, and reproduction in any medium, provided the original work is properly cited.
License URL: https://creativecommons.org/licenses/by/3.0/

==============================
Miami ***Nature Biotechnology*** Short Reports
***TheScientificWorld*** (2001) 1(S3), 105SR
ISSN 1532-2246; DOI 10.1100/TSW.2001.119

### DETERMINATION OF THE EFFECTS OF CAMPTOTHECIN ON CELL CYCLING AND APOPTOSIS IN HUMAN LEUKAEMIC CELL LINES

Anne Cox[*], Dawn A. Bradbury[#], Tracy D. Simmons, Kevin J. Slater, and Sharon P.M. Crouch

LumiTech (UK) Ltd, Nottingham Business Park, City Link, Nottingham NG2 4LA, UK
[#]Clinical Sciences, Nottingham University, Nottingham, UK
[*] a.cox@lumitech.co.uk

**INTRODUCTION.** The measurement of ATP is a recognised method for determination of proliferation or the effect of toxic environments on the cell. More recently, the relationship between ATP and ADP has been shown to be an important factor in determining the apoptotic or necrotic state of the cell (1). The ratio of ADP to ATP shows significant correlation with the degree of apoptosis, as measured by the estimation of the sub-$G_0$ by propidium iodide staining of the nuclei and flow cytometry. Camptothecin is a topoisomerase inhibitor and as such, has effects on the cell cycle prior to inducing apoptosis. This compound has also been used as a chemotherapeutic agent in the treatment of a number of malignancies including leukemias. We have investigated these effects in a number of human leukaemic cell lines, concentrating on U937 and HL-60 cells, together with blast cells isolated from the peripheral blood of patients with acute myeloid leukemia.

**METHOD.** HL-60 cells were incubated for 4 hours, while with the U937 cells a 24-hour incubation was required for induction of apoptosis, with increasing concentrations of camptothecin. The AML blast cells, from 10 patients were incubated with increasing concentrations of camptothecin, Ara-C and etoposide for 4 to 24 hours. The extent of apoptosis was shown by measurement of the ratio of ADP:ATP using the ApoGlow Kit (LumiTech (UK) Ltd). The presence of apoptotic events within the cell population was confirmed using Annexin V, JC-1, and propidium iodide (PI) staining and flow cytometry. The PI staining was also used to determine the relative percentage of cells in different phases of the cell cycle.

**RESULTS.** The HL-60 and U-937 cells showed a concentration dependent effect on apoptosis induction with concentrations of camptothecin from 100 to 500nM. At the highest concentration of camptothecin used (1000nM) there was a significant reduction (n=7, $p<0.0001$) in the ratios compared with the 500nM sample. Comparison with the PI data revealed emergence of the G0/G1 peak showing that a subpopulation of cells was beginning to cycle again. With HL-60 cells at 1000nM of the drug, there was an increase in the percentage viable cells as shown by exclusion of PI, this correlated with a reduction in the level of ADP relative to ATP with the emergence of a resistant viable population. Of the AML cells investigated there was a marked difference in the response of the patient cells to the different drugs investigated, with respect to induction of apoptosis and drug resistance.

**DISCUSSION.** The U937 cells displayed variations in responses according to the concentration of camptothecin used. The cells arresting in S-phase at low concentrations, could be detected by an increase in ATP and a relative decrease in the amount of ADP, when compared to control cells. Cells were then pushed into apoptosis from S-phase (2,3), as seen with the higher concentrations of drug. At the highest concentration, the re-emergence of G0/G1 showed that, while the majority of cells had succumbed to the drug through apoptosis and then secondary necrosis, there was a small population of resistant cells that had begun to proliferate. The use of this rapid, sensitive bioluminescence assay has the potential to be used as a screen for determination of drug resistance, in a number of different malignancies. It also lends itself well to being used as a tool for determining the efficacy of chemotherapy for individual patients.

**REFERENCES.**

1. Bradbury, D.A., Simmons, T.D., Slater, K.J., and Crouch, S.P.M. (2000) J. Immunol. Methods 240, 79-92
2. Ferrero, C., Quemeneur, L., Fournel, S., Prigent, A.F., Revillard, J.-P., and Bonnefoy-Bernard, N. (2000) Cell Death Differ. 7, 197-206
3. Goldwasser, F., Shimizu, T., Jackman, J., Hoki, Y., O'Connor, P.M., Kohn, K.W., and Pommier, Y. (1996) Cancer Res. 56, 4430-4437
